# Emerging pollutants in the EU: 10 years of NORMAN in support of environmental policies and regulations

**DOI:** 10.1186/s12302-018-0135-3

**Published:** 2018-02-22

**Authors:** Valeria Dulio, Bert van Bavel, Eva Brorström-Lundén, Joop Harmsen, Juliane Hollender, Martin Schlabach, Jaroslav Slobodnik, Kevin Thomas, Jan Koschorreck

**Affiliations:** 1 INERIS, National Institute for Environment and Industrial Risks, Verneuil-en-Halatte, France; 2 NIVA, Norwegian Institute for Water Research, Oslo, Norway; 3 IVL, Swedish Environmental Research Institute, Stockholm, Sweden; 4 Wageningen Environmental Research, Wageningen, The Netherlands; 5 Eawag, Dübendorf, Switzerland; 6 NILU, Norwegian Institute for Air Research, Kjeller, Norway; 7 Environmental Institute, Kos, Slovakia; 8 UBA, Federal Environment Agency, Dessau-Roßlau, Germany

**Keywords:** NORMAN network, Emerging substances, Contaminants of emerging concern, Science-to-policy, Environmental monitoring

## Abstract

In 2005, the European Commission funded the NORMAN project to promote a permanent network of reference laboratories and research centers, including academia, industry, standardization bodies, and NGOs. Since then, NORMAN has (i) facilitated a more rapid and wide-scope exchange of data on the occurrence and effects of contaminants of emerging concern (CECs), (ii) improved data quality and comparability via validation and harmonization of common sampling and measurement methods (chemical and biological), (iii) provided more transparent information and monitoring data on CECs, and (iv) established an independent and competent forum for the technical/scientific debate on issues related to emerging substances. NORMAN plays a significant role as an independent organization at the interface between science and policy, with the advantage of speaking to the European Commission and other public institutions with the “bigger voice” of more than 70 members from 20 countries. This article provides a summary of the first 10 years of the NORMAN network. It takes stock of the work done so far and outlines NORMAN’s vision for a Europe-wide collaboration on CECs and sustainable links from research to policy-making. It contains an overview of the state of play in prioritizing and monitoring emerging substances with reference to several innovative technologies and monitoring approaches. It provides the point of view of the NORMAN network on a burning issue—the regulation of CECs—and presents the positions of various stakeholders in the field (DG ENV, EEA, ECHA, and national agencies) who participated in the NORMAN workshop in October 2016. The main messages and conclusions from the round table discussions are briefly presented.

## Background

This paper, triggered by the 10th anniversary of the NORMAN Association [1] and the outcomes of the workshop that was organized to mark this occasion, discusses the work performed within the NORMAN network over the last 10 years and the way forward to improving Europe-wide collaboration on CECs and related policy-making.

It is now about a decade, since the term “contaminants of emerging concern” (CECs) became a common term for chemicals that are currently not regulated (not submitted to a routine monitoring and/or emission control regime), but may be under scrutiny for future regulation. In addition, it is now common knowledge that the contaminants of interest are not necessarily newly developed chemicals: most CECs are substances that have entered the environment for years, even decades, but their presence has only recently begun to be investigated. Most known CECs include industrial compounds, pharmaceuticals, personal-care products, biocides, and plant protection products, but the list of compounds is constantly growing and this is not surprising when we consider that more than 100 million chemical substances are currently registered in the Chemical Abstracts Service (CAS) and about 4000 new ones are registered every day. According to the number of registered and pre-registered substances in REACH, 30,000–50,000 industrial chemicals are found in daily-use products [2, 3] and they are potentially ultimately released into the environment. Chemicals are everywhere, in the water we drink, in the food that we eat in the homes in which we live.

There are increasing concerns about the combined effects of this multitude of chemicals as they enter the environment and the food chain, although each chemical used in a minute quantity may be considered harmless [4, 5]. In addition, there is today a general consensus among policy-makers that emerging substances need to be addressed in a systematic and coherent manner. It is also widely accepted that there is a need for an early warning system able to play the role of the “watchdog”. Such a system should anticipate the risks associated with the dynamic change in the use of chemicals so as to prevent the environmental impact of chemical substances before they become “contaminants of emerging concern”. In other words, our ultimate objective should be to advance our knowledge and environmental monitoring abilities to the point, where the need for the term “emerging” disappears altogether [6].

In 2004, NORMAN came into existence following a call by the EU Commission (DG Research) to create “a permanent network of reference laboratories and related organizations dealing with emerging environmental substances” [7]. Its main objectives—on which it has been working actively over the past 10 years—are to improve the exchange of information on emerging substances and to foster harmonization of protocols and improvement of data quality.

Today, NORMAN is an independent and highly recognized network of reference laboratories, research centers, and related organizations for the monitoring of contaminants of emerging concern. In 2006, its first full year of operation, it was a consortium of 17 members; today, it is a self-sustaining, non-profit organization of more than 70 members.

Five pillars constitute the NORMAN objectives:

- Independent, transparent, and open network, working for a sustainable environment without harmful substances.
- Go-to organization for issues on emerging substances in the environment.
- Watchdog and alarm bell for emerging environmental threats.
- Bridge between science and policy-making.
- Platform for innovative bottom–up initiatives to explore new monitoring challenges.

**Fig. 1:**
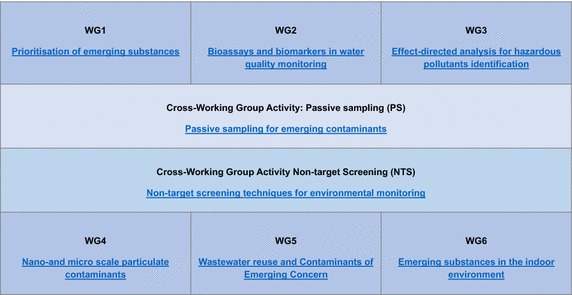
Overview of the NORMAN Working Groups

To achieve these goals, NORMAN brings together not only the scientific community on emerging substances but also the many agencies actively involved in the decision-making on emerging substances and even private companies. The multidisciplinary membership of NORMAN has proven to be a strong point as it helps to pull knowledge of emerging substances together and pushes the latest scientific findings towards policy-making. The activities are organized in eight working groups dealing with different CECs aspects, i.e., prioritization, effect-based tools, effect-directed analysis, nanomaterials, wastewater reuse, indoor environment as well as two cross-working group activities on passive sampling and non-target screening (Fig. 1).

NORMAN publishes a bulletin on CECs, with information on current initiatives, results of collaborative projects and future perspectives in the field [8]. More than 30 international events [9] have been organized by NORMAN since 2006 and position papers have been published on various relevant topics by NORMAN experts, such as passive sampling [10], effect-directed analysis [11], and more recently a paper with 10 recommendations [12] for the review of the Water Framework Directive (WFD) [13], developed in collaboration with the SOLUTIONS project [14].

The stakeholders at the 10th anniversary workshop unanimously agreed that NORMAN has succeeded in building a strong infrastructure and developing tools to connect science and policy. NORMAN has also proven to be a good platform to reach consensus among experts for harmonization of practices.

Overall, NORMAN aims to play the long-term role of global early-warning platform for CECs, closely related to the non-toxic environment strategy [15].

## NORMAN achievements and future perspectives

A first consideration when speaking about NORMAN’s achievements over the last 10 years is that NORMAN has been able to establish and operate a collaboration mechanism to deal with the following crucial questions about emerging substances: What are the most suitable techniques and strategies to identify and prioritize potential problematic chemicals? Do we have enough data to assess the risks associated with CECs? Do the data pass quality criteria and are they representative enough? And do we have access to all the data that are available?

### Prioritization of CECs

**Fig. 2:**
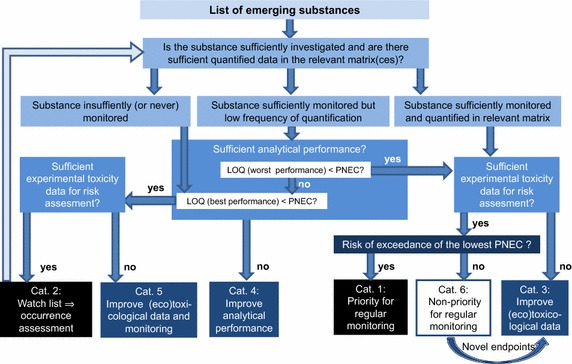
Prioritization scheme of the NORMAN network

The prioritization of chemical contaminants is a task of primary importance for environmental managers and the scientific community, for the definition of priority actions for pollution prevention and control, and the efficient allocation of resources to address current knowledge gaps. Starting from the observation that, for most emerging substances, it is primarily the knowledge gaps which still prevent proper risk assessment and risk ranking, NORMAN has developed a rational and holistic prioritization approach (Fig. 2) which gives more systematic consideration to the knowledge gaps relating to emerging substances [16, 17]. The scheme has been used by NORMAN to provide recommendations to the European Commission for the prioritization of the compounds on the first European Watch List [18] and has also been adopted by regulatory agencies in France [19, 20] and in Slovakia [21].

In the light of the experience acquired, NORMAN is now committed to the further development of the current scheme with the extension of the original NORMAN list of substances (ca. 900 compounds) to a much larger list of several thousands of compounds. This goes along with the establishment of dynamic links with existing databases (such as, for example, the US EPA CompTox Chemistry Dashboard [22]) for a more powerful and systematic retrieval of supporting data for prioritization of substances, and the introduction of new indicators for better integration of the results from novel monitoring-based approaches, such as suspect and non-target screening (NTS) as well as effect-based methods (EBM) in the prioritization process. NORMAN fosters an integrative approach for the prioritization of CECs [23], which relies on three pillars: the first is EMPODAT [24], a powerful database system which has been developed to store the monitoring data collected by NORMAN members and as a tool for use by regulators and scientists alike for the prioritization of CECs. Its added value will be further increased in the future thanks to its full integration into the European Information Platform for Chemical Monitoring (IPCHEM) [4], which will improve systematic exploitation of raw monitoring data to support prioritization exercises. This is closely connected with the second pillar, the EMPODAT ECOTOX module, a platform for systematic collection and evaluation of the relevance and reliability of ecotoxicity studies which aims to become an essential tool for the European community of ecotoxicologists for the derivation and harmonization of predicted no-effect concentration (PNEC) threshold levels. NTS, the third pillar, includes recent workflows for the application and evaluation of high-resolution mass spectrometry for identification of suspects and unknowns. The results from NTS, i.e., checking presence/absence and semi-quantitative information about these compounds in a large number of samples by NORMAN partners in different countries, will help in the future to prioritize the most relevant compounds for possible further evaluation as substances of potential regulatory concern. The collaborative NormaNEWS [25] joint activity has already successfully demonstrated the usefulness of the retrospective screening of high-resolution mass spectrometric data in establishing the spatial and temporal occurrences of newly identified compounds of potential emerging concern [26].

### Data collection and data management

Reliable identification and prioritization of relevant CECs is strongly dependent on the quality and quantity of archived monitoring data. The development of databases such as EMPODAT and improvement of data exchange have been NORMAN’s core business since the start of the project. A clear need was identified at the time of the launch of the NORMAN project, which was confirmed by the first NORMAN Databases workshop in 2011 [27], where experts concluded that, in spite of the numerous chemical monitoring activities carried out in the EU and worldwide and the significant amounts of data generated by the scientific community within research projects, environmental monitoring data were not systematically collected at the EU level [28]. EMPODAT [24] is today the largest database on emerging substances worldwide, with about ten million data records for more than 500 emerging substances. The interest and contribution of the network partners have enabled the database system to constantly grow, and new modules for accommodation of passive sampling, indoor environment, bioassays monitoring, antibiotic-resistant bacteria (ARBs), and antibiotic-resistant genes (ARGs) data are now under development. Besides that, the ECOTOX module already contains predicted and experimental PNECs for more than 13,000 substances and its sustainable growth is one of the priority tasks of the NORMAN network. The trend is clearly towards encouraging data sharing, improving access and use of available data along with improvement of their quality. The value of the NORMAN platform is fully recognized by the European Commission, with which NORMAN has recently started a close collaboration to achieve permanent integration of EMPODAT in IPCHEM [4].

### Evolution towards “big data” management: from hundreds to tens of thousands of candidate substances

We are increasingly aware that there is a need to evolve towards a system able to deal with several thousands of compounds which may enter the environment. NORMAN is already working to replace the original “NORMAN list of emerging substances” of about 900 compounds with a much larger list of substances. The NORMAN Suspect List Exchange database (SusDat) database [29] has recently been launched and already includes more than 40,000 compounds as a common effort of European and North American researchers. All suspect lists currently available in SusDat can be viewed at the NORMAN website and are being progressively integrated into the US EPA CompTox Chemistry Dashboard [22]. This large list will become the new “universe” of compounds for prioritization, and the NORMAN List will be defined as the list of top priority compounds in each prioritization action category.

Non-target screening analysis, combined with the integration of high-performance computing, becomes “ready to go” for environmental applications [30] and moves traditional exposure analysis to ‘big data’: the NORMAN ‘Digital Sample Freezing Platform (DSFP)’ is currently under development to host in a harmonized format full-scan high-resolution mass spectrometry (HR-MS) data, allowing for high-throughput processing (including retrospective analysis) of any environmental sample for a wide range (thousands) of pollutants. The concept of collaborating in one DSFP and sharing its ‘big data’ has been recently tested among a core group of NORMAN, with data sets obtained within the Joint Danube Survey 3 (surface water samples) [31] and the EU/UNDP EMBLAS project (marine water, sediment, and biota samples) [32]. Further improvement of functionalities of the DSFP (upload of raw mass chromatograms, visualisation of data, batch mode processing, use of MS–MS information, etc.), the extension of its functionalities for archiving and processing of gas chromatography–HR-MS data and testing of various options for archiving and processing of ‘big data’ at the wider European scale are planned for 2018 and beyond.

### Methods’ harmonization and validation

The NORMAN community is recognized as particularly strong in analytical matters and the studies organized by the network represent a crucial step for the scientific community and for environmental agencies in the preparation of the ground for validation and harmonization of innovative sampling and monitoring tools before their possible future implementation in regulations.

As regards improvement of data quality, one major achievement of NORMAN has been the development of a common framework for validation of chemical and biological monitoring methods—a protocol which is now adopted as a Technical Specification (TS) of the European Committee for Standardization (CEN) (CEN TS 16800:2015) [33, 34]. In other words, NORMAN has defined a clear list of “rules” that the laboratories need to observe to be able to state that their method is “validated”—and it is well known how method validation is crucial, especially when it comes to the measurement of substances which laboratories are not familiar with, with clear consequences for the quality and reliability of the results produced. Besides that, NORMAN has organized interlaboratory studies on substances of priority interest in research [35, 36] and has more recently extended these inter-comparison activities to passive sampling [37], bioassays [38], and non-target screening methods [31].

### New tools to improve future monitoring and regulation of CECs

#### Non-target screening

In line with the strong expertise of the NORMAN network in the field of high-resolution mass spectrometry techniques and NTS approaches, several activities have been launched over the past years and continue to be promoted to improve harmonization of liquid chromatography coupled to high-resolution tandem mass spectrometry [LC–HR-MS(MS)] and gas chromatography coupled to mass spectrometry (GC–MS) NTS protocols, in connection with the use of structure elucidation and pollution pattern recognition tools. Besides the Suspect List Exchange database [29] and the “Digital Sample Freezing Platform” [39], the NORMAN MassBank database [40, 41] was created in 2011 as an open-access database of mass spectra which now contains spectra of more than 1000 environmental contaminants to support the identification of “unknowns” (i.e., compounds with an unidentified chemical structure). A Collaborative Trial (CT) was organized for the first time worldwide in 2013 to study laboratories’ common practices and promote harmonized terminology, workflows, and reporting formats for the use of non-target and suspect screening in the area of environmental analysis [31]. Another key action was the development of a harmonized model for the prediction of the retention time index (RTI) for NTS and retrospective analysis of a large number of potential emerging substances [42, 43]. The NORMAN RTI has already been incorporated into the DSFP and it is expected that it will also soon be included in the open mass spectral libraries such as MassBank [40], STOFF-IDENT [44], and related platforms (e.g., FOR-IDENT [45]).

#### Effect-based tools

Bioassays are the only currently available methods able to respond to the recently recognized need to address unknown mixture risks present in the water bodies, which can then be related to specific chemical compounds via chemical analysis: instead of measuring a limited list of target individual substances known to be responsible for a given effect, it makes more sense to measure all substances (target substances plus other unknowns) that may contribute to that effect [46]. The EU Water Directors recently supported the proposal by the Commission to consider such a more holistic approach for regulation of chemicals in the aquatic environment in view of the WFD review [47] and an effect-based methods (EBM). Activity was launched as part of the CIS-WFD Programme in 2017 [48]. The successful introduction of these tools in environmental monitoring programs in the future will, however, depend on the successful transition from the current system to a new European framework defining the performance criteria for the selection of bioassays to be applied, and the QA/QC criteria for validation of the results obtained with these new methods, the effect-based trigger (EBT) values necessary for the interpretation of the data, and the way to proceed when an EBT is exceeded. NORMAN is actively contributing to this process, helping the construction of a common position of the European experts on the use of bioassays in the regulatory framework of the WFD and, more recently, in the drafting of the EU policy instrument for Water Reuse. Besides the interlaboratory study organized in 2009 to assess the comparability of results obtained with a battery of bioassays [38], NORMAN has contributed to the estrogen-monitoring project which has recently provided concrete demonstration data about the performance of the tested effect-based methods [49]. A comprehensive in-depth overview of effect-directed analysis (EDA)—the approach of choice to provide information on the compounds causing the observed effects—has been published by the respective NORMAN Working Group to meet the increasing demands for its most efficient application [11].

NORMAN supports the implementation of effect-based monitoring tools in water-quality assessment [50]. The integration of effect-based tools and ‘comprehensive’ NTS techniques has the potential to result in a more robust identification of priority CECs. In this context, EDA may be established in the future as part of the protocol to be applied at the sites where effect-based trigger values are exceeded. As an advanced screening tool, instead of time-consuming fractionation followed by effect tests and NTS, effect-based results and NTS data of whole samples can be integrated via the application of multivariate analysis (virtual EDA approach), to find correlations between effects and typical contamination patterns [43].

#### Passive sampling

NORMAN promotes the use of passive sampling tools, *inter alia* to address the current lack of temporal representativeness in water body monitoring and as a supplement to biota monitoring [10, 51].

The interest of NORMAN in passive sampling techniques started as early as 2009 with the organization of a large international interlaboratory study to assess the applicability of passive sampling for the monitoring of several groups of emerging aquatic pollutants, including pharmaceuticals, pesticides, steroid hormones, brominated diphenyl ethers, and PFOA/PFOS [10, 37]. The study showed that the passive sampling process caused less variability in results than the laboratory analysis and the translation of passive sampling data into water concentrations. A need was identified for improving the accuracy of analysis and calibration of adsorption-based passive samplers, as well as for more confidence in practical application of partition-based passive samplers.

Further actions were then organized by NORMAN [52–54] to investigate how environmental quality standards (EQS) values relate to results obtained from passive sampling and vice versa and to clarify where passive sampling could fit into the schemes that are currently applied for assessment of the chemical and ecological status of water bodies under the WFD [51].

Today, it is well recognized that there is a strong potential to use passive sampling tools for regulatory purposes, in particular as regards the use of these devices in concert with chemical monitoring in biota to support the chemical status assessment in European water bodies [54, 55].

To increase the relevance of passive sampling in this context, data sets based on concurrent passive sampling and biota monitoring are strongly needed. Such data sets may need to be developed at the European level and there is an opportunity for NORMAN members to contribute to federating national on-going initiatives (such as the large demonstration project organized by AQUAREF in France in 2018–2019), to similar studies in other European countries. This would facilitate the knowledge exchange and harmonization of methodology for better comparability of data at European scale.

To allow the use of passive sampling data for regulatory monitoring, it is also important to prepare the basis for archiving the generated data in appropriate databases in a harmonized format. Here, the contribution of NORMAN experts has resulted in harmonized guidelines for reporting of data obtained by passive sampling tools [NORMAN Data Collection Templates (DCTs)], which is expected to facilitate the wider exchange of monitoring data obtained with passive samplers [43]. Based on these standardized DCTs, a prototype online database module for passive sampling data has recently been developed and tested with JDS3 data [56] within the SOLUTIONS project [57].

Additional perspectives arise when considering the opportunities offered by the combination of passive sampling and non-target and suspect screening procedures. Relatively little work has been undertaken in this area until now. A suitable choice of polymer and extraction protocol can enable the scientist to pre-concentrate chemicals from a complex matrix while leaving behind a significant proportion of unwanted matrix affecting the performance of the analysis. This is especially relevant for complex matrices such as sediments, sludge, or biological matrices. Passive sampling of air, sediments, and water is amenable to non-target approaches, and novel applications for sampling of biota [58, 59] or to further our understanding of the human exposome are highly promising [60]. NORMAN, through its cross-working group activities on passive sampling and non-target screening, is ideally suited for leading this work.

### Other areas of concern that NORMAN is exploring

#### Nano- and micro-scale particulate contaminants

The steeply increasing production volumes of engineered nanomaterials as well as incidental and natural particulate contaminants will eventually lead to a proliferation of these materials in the environment, with poorly understood effects on ecosystems. NORMAN aims to contribute to increased understanding of particle behavior in the environment and the resulting consequences for ecosystems.

To that end, NORMAN activities address the fate and transformation of particulate contaminants in natural (e.g., freshwater, floodplains, and marine systems) and technical (wastewater treatment and sewage treatment) systems. NORMAN will keep working to develop analytical methods (including sampling, sample preparation, e.g., particle extraction, clean-up, and analytical tools to detect, quantify, and characterize particulate contaminants in complex matrices) [61]. Finally, NORMAN will contribute as a platform facilitating access to research infrastructure and promoting exchanges of methods and materials.

In 2016, the NORMAN members decided to add microplastics as a new issue under the scope of the NORMAN activities [62]. NORMAN expertise in, e.g., data management, method development, and harmonization is expected to contribute to improve the assessment of plastic particles in the environment.

#### Wastewater reuse

A series of actions are currently being taken by the Commission to promote the reuse of treated wastewaters, including a legislative proposal on minimum requirements for reused water, e.g., for irrigation and groundwater recharge [63]. However, a number of questions are still open and they are crucial to prevent and manage health and environmental risks. Important challenges are, amongst others, associated with the presence of non-regulated contaminants, whose environmental fate and long-term effects are not yet fully understood. Moreover, the threat posed by the spread of antibiotic-resistant bacteria and the multiple evidences that domestic wastewater is amongst their major environmental reservoirs raise key questions that the scientific community is committed to answer. Today, there is a consensus that reclaimed wastewater releases antibiotic-resistant bacteria and their genes. There is, therefore, an urgent need for better understanding of the presence and fate of micro-contaminants promoting the widespread of antibiotic-resistant bacteria and genes in wastewater treatment plant (WWTPs) effluents before their disposal or further reuse [64].

In response to these needs, a new NORMAN activity kicked off in 2013 [65] with the commitment to work on: (1) the definition and establishment of a harmonized protocol for measurement of antimicrobial resistance; (2) the development of a European database to compile information on the overall abundance and diversity of different genetic determinands in wastewater effluents and receiving environments; and (3) the drafting of recommendations to the European Commission [64].

Two screening campaigns of selected ARGs were organized in 2014 and in 2015 on a representative set of WWTPs around Europe and Mediterranean countries [66]. Besides the contribution of these campaigns to the assessment of differences in the abundance and diversity of ARGs over distinct municipal wastewater treatment plants and geographic areas, a major follow-up of this study was the on-going work of the NORMAN experts on the definition of a harmonized protocol and interlaboratory calibration criteria to support a reliable ARG quantification. This is essential to assess the degree of ARG occurrence and environmental contamination, and it has never been done before. Currently, there is no baseline on the prevalence of resistance genes in aquatic (natural) environments and, to obtain this baseline, standardized protocols are pivotal. Such a baseline and a better process understanding (and corresponding models) will help to assess the potential risk of antibiotic resistances in the aquatic environment and water reuse.

Through its activities and collaborations with other relevant EU-funded projects (NEREUS [67] and ANSWER [68]), NORMAN developed in 2017 a new Data Collection Template used as a basis for a new EMPODAT database module concerning ARBs and ARGs which will be fed by this project.

#### Indoor environment

There is potential for extending the scope of NORMAN activities to other environmental matrices and compartments (air, sediments, biota, etc.). Indoor environment appears as a relevant key domain for NORMAN’s missions when looking at the concerns associated with emerging contaminants in human matrices. Articles and consumer products used indoors may contain a variety of both well-known chemicals and emerging substances [69–71]. Chemicals are emitted in the indoor environment and indoor air and dust is an important pathway of chemical exposure for humans. A new NORMAN activity for the indoor environment was launched in 2014 aimed at identifying CECs for the indoor environment and at storing respective data in a harmonized way in EMPODAT. Measuring goes along with prioritization of relevant compounds in the indoor environment, the identification of emission sources of CECs, and relevant exposure pathways. The ultimate goal of this working group is to raise awareness of CECs in indoor environments and possibly to contribute to development of new EU legislation regulating the occurrence of CECs in the indoor environment [72].

A workshop on “Emerging pollutants in non-industrial indoor environments” was organized in June 2015 at NILU, Norway to mark the first actions of NORMAN in this field [72]. Further to the workshop various activities have already been organized by NORMAN in the field of CECs in the indoor environment.

A collaborative trial on non-target and suspect screening of indoor dust was launched in 2016 for the identification of pollutants specific to indoor environments, which provides relevant input for harmonization of practices and for the definition of a list of CECs relevant for indoor environment and their prioritization. Strategies for prioritization of CECs indoors are currently being discussed and a subgroup for this task has recently been formed, in connection with the already operational NORMAN Prioritization Working Group.

The generation of high quality and comparable monitoring data—still scarce and highly scattered in the indoor environment—and minimum quality requirements for their harmonized storage in a common database is crucial to support prioritization activities. Thanks to NORMAN activities, a new Data Collection Template with relevant metadata for indoor air and dust has been developed for the indoor environment module of the NORMAN EMPODAT database.

Finally, NORMAN is committed to improving harmonization of sampling protocols for dust and air. The NORMAN indoor environment working group made a first inventory of sampling protocols used to collect indoor dust and air. The use of different sampling protocols can result in different particle size fractions collected and hence in differences in concentrations of SVOCs. There is, therefore, a great need for an inter-comparison study of different dust sampling protocols, and the setting-up of a comparison study within NORMAN for sampling protocols of dust has been proposed for 2018.

## Stakeholders’ views and recommendations for the NORMAN network

The NORMAN Steering Committee organized on the 10th anniversary of the network [73] a stakeholder workshop, which took place in Brussels on 26 October 2016. It attracted about 90 participants, with representatives from 60 organizations, including the European Commission, European Chemicals Agency (ECHA) and European Environment Agency (EEA), national authorities, research centers, academia, industry, and international stakeholder organizations.

National and European agencies, the European Commission, and relevant stakeholders were invited to present their experience with the work performed by the network so far and give their recommendations about NORMAN’s future roadmap, with a view to improving Europe-wide collaboration on emerging pollutants and policy-making. The workshop included two panel discussions.

### EU Commission DG ENV

According to DG ENV, the tools developed by NORMAN are useful to the Commission services and to the Member States. NORMAN has contributed significantly to the European prioritization process of the WFD with unique data sets (15% of the monitoring data used in the review process for the EU priority substances have been retrieved from the NORMAN EMPODAT database). However, challenges still remain for representative monitoring data of sufficient quality and for more holistic monitoring approaches. J. Romero (DG ENV) highlighted the following four main challenges.

As regards river basin-specific pollutants (RBSP), the efforts of the Member States clearly indicate that there is a need for improved and more comparable approaches between countries, in terms of both identification and monitoring of RBSP.

Prioritization relies largely on sound and comprehensive monitoring data. It is widely recognized that the lack of data is the primary cause of the lack of regulation of CECs, as a result of the vicious circle where: “no monitoring means no data, and no data means no regulations”. The Commission action to break this vicious circle was the introduction of the EU watch list [18] for a short list of selected compounds. In addition, the Commission introduced IPCHEM [4] to collate monitoring data from the environment and human populations and to make these data accessible for regulation, research, and the public.

Although monitoring data for regulated substances and emerging contaminants will increase in numbers and be more accessible in the future, the question remains whether we are addressing chemical pollution in the environment in a sustainable and efficient manner. There is the impression of playing catch-up, as there is not yet an established mechanism to anticipate the challenges of the future. Effect-based tools, non-target screening techniques, passive sampling, effects directed analysis, etc. are new and promising options for future routine use in chemicals and water management.

However, it needs to be ensured that novel monitoring tools are appropriate for regulatory programs. The extra benefit of novel tools needs to be demonstrated and common harmonized practices need to be agreed upon by environment agencies before they can be written into the regulations. NORMAN has a clear role here in facilitating the transfer from science to policy. NORMAN can play an important role in the Common Implementation Strategy (CIS) of the WFD [18], in particular in bringing fresh ideas and testing of new tools to improve future strategies for water-quality monitoring.

### National agencies: two examples

#### NORMAN in support of environmental legislation

The feedback from AFB in France was that NORMAN helps water managers. The added value of NORMAN for national activities is that NORMAN draws together expertise from across the EU and beyond, and promotes synergies across research teams: this adds significant value to the CIS in support of the WFD. Furthermore, NORMAN’s strategic focus and its ability to help expertise and data sharing stimulate the development of complementary national R&D strategies.

In France, national authorities adopted NORMAN products to develop the national strategies for water management. The mechanism currently used in France for the national review of the list of River Basin-Specific Pollutants (RBSPs) and the launch of regular screening studies on emerging substances is based on the principles of the NORMAN prioritization scheme.

In this context, a dedicated “prospective” surveillance network has recently been established, which will also involve innovative tools NTS, bioassays and passive sampling, building upon the results of the NORMAN interlaboratory studies, recommendation papers, etc. The French case study is a demonstration of how EU member states and/or the EU can benefit from NORMAN activities with regard to science-to-policy links.

The view of AFB is that NORMAN goes beyond networking scientists and research institutes. It also involves regulatory agencies and industry. Thanks to this tripartite nature, the NORMAN community is aware of the requirements and challenges faced by water managers in the implementation of the current legislation and the necessary steps for the implementation of innovative tools.

#### The need for monitoring data in chemicals legislation

The positive impact of NORMAN as regards the generation of environmental occurrence data was addressed by the German Environment Ministry with reference to the case of biocides in the aquatic environment and the need to improve the legislation for safe marketing of biocidal products. In 2016, NORMAN organized a workshop on environmental monitoring of biocides in Europe [74]. Monitoring data can tell us whether there are shortcomings in the authorization procedure, and whether risk mitigation measures are designed in a reasonable manner. They can also serve as a means to better focus surveillance and control measures.

As yet, far less data for biocides in the environment are available in comparison with plant protection products and pharmaceuticals. New data can create pressure on policy makers for a level playing field in, e.g., regulating biocides and plant protection products with equivalent protection goals. The Directive 2009/128/EC on the “sustainable use of pesticides” [75] adopts an overarching approach to reduce the overall risks and impacts of pesticides on environment and health. The German Environment Ministry emphasized that new monitoring data for biocides can help to achieve a protection level comparable to the sustainable use law for plant protection products.

### International Commission for the Protection of the Danube River

The International Commission for the Protection of the Danube River (ICPDR) stated that there is an established fruitful cooperation between the NORMAN network and the ICPDR, tested most recently on the case study of the third Joint Danube Survey (JDS) which is organized every 6 years (2001, 2007, and 2013) by the ICPDR’s 14 member countries and the EU.

In JDS3 (2013), the monitoring involved a number of new techniques provided by the NORMAN network in synergy with the FP7 SOLUTIONS research project, including effect-based screening using large-volume solid-phase extraction, target, suspect and non-target screening of hundreds of organic pollutants using high-resolution mass spectrometric techniques and new passive sampling approaches to detect trace concentrations of CECs.

The prioritization methodology developed by NORMAN, which has been presented to the Monitoring and Assessment Expert Group of the ICPDR, was applied to the JDS3 results and produced a list of 20 pollutants suggested as relevant for the Danube River Basin. These substances were presented in the second Danube River Basin Management Plan published in 2015.

The above cooperation was proclaimed as a unique example of science-to-policy action in a wide European context. The next JDS is planned for 2019 and it is already foreseen that it will include large-scale analysis of CECs as well as non-target screening in surface, ground, wastewater, and biota samples.

### Regional Seas Conventions and the Marine Strategy Framework Directive

The Regional Seas Conventions and the Marine Strategy Framework Directive recommended that NORMAN should take an active role in the discussions about CECs in the marine compartment. This would involve active support of NORMAN experts in the Regional Sea Conventions, non-EU Partners in shared marine basins and in the Marine Strategy Framework Directive (MSFD) [76] to define a list of compounds of sub-regional concern. According to the stakeholder, NORMAN should also have a role in ensuring improvement of the ability of laboratories to achieve quantification limits (LOQs) in line with toxicologically relevant concentrations in the marine environment.

The importance of NTS techniques for the monitoring of chemical contaminants in the marine environment was also stressed. NTS will provide major changes in policy options, but further collaboration is still required for its implementation. NORMAN should have a role in providing an independent review and support for the development and implementation of innovative techniques in the marine environment.

Finally, there is a need for a repository of geo-referenced harmonized marine data on emerging substances from scientific publications and projects.

## Key recommendations

The discussions between scientists and stakeholders involved in policy-making brought up several key recommendations for NORMAN to continue its activities.

Identification and regulation of emerging substances consist of many challenges for policy and research. They include population growth and an ageing population, climate change, new materials, new technologies, and the circular economy. The vision for future chemicals policy is that pollutants should be dealt with in an integrated manner in an overarching chemicals policy framework covering all types of chemicals and all uses, beyond the current sector-specific regulations.

Monitoring data are established indicators in water and chemical management. They are used to safeguard the effectiveness of environmental policy and to trigger new regulatory actions. However, for many substances, there are no—or only insufficient—monitoring data. New analytical techniques such as NTS are likely to generate much more chemical monitoring data in the future. A wider picture of contaminants in the environment will become a challenge for environmental legislation, e.g., the WFD and chemical legislation such as REACH and the overarching Commission’s 2018 Strategy for a non-toxic environment [15].

NORMAN is establishing collaborations with EU regulatory bodies, e.g., ECHA. A reliable assessment of chemicals of emerging concern in environmental media requires exposure data to be linked with information on marketed substances. Following this line, it is important to establish a mutual data exchange between NORMAN databases and IPCHEM. Some of the data that have been generated (e.g., produced by research studies) are still kept in databases with restricted access, so that they cannot be used for assessment of occurrence levels. For data produced with public funds, there should be a mechanism to make them available to the public authorities and institutional bodies by default. Furthermore, the example of per- and polyfluorinated alkyl substances (PFAS) indicates the need to look beyond the list of registered substances: only 5% of PFAS have a CAS Registry Number as a unique, unmistakable identifier for chemical substances. Novel analytical techniques such as NTS can identify the presence of these compounds in the environment.

There is still a need to better investigate individual chemicals of emerging concern and their transformation products by developing analytical methods to determine occurrence of these compounds in the environment; understanding how they may be released or formed; and identifying their potential environmental effects. For example, more research and more monitoring data may be needed for persistent and mobile organic compounds, i.e., PMOC: they are difficult to remove in WWTPs and they can, therefore, be seen as relevant emerging contaminants in the aquatic environment. However, the present process of ranking and selection of priority substances and setting of EQS does not adequately address persistent and mobile substance properties. They should, therefore, be given a higher score in the priority substances selection process to be consistent with the WFD Art. 7, protection objectives, as has been recently proposed by [77].

NORMAN has proven to be an efficient platform for new monitoring approaches. NORMAN brings together expertise from leading research groups and is consequently a reservoir for innovative initiatives. NORMAN will further promote the use of both environmental data (chemical concentrations) and biomonitoring data (data from bioassays). There is already clear evidence from recent research studies of the added value of effects based tools, e.g., in the assessment of estrogenicity [78–80]. In the future, the range of endpoints should be broadened to enable wider application in monitoring. This should involve a systematic approach for grouping of chemicals in accordance with their mode of action, use sector, etc., as the ‘individual substance’ regulation is not efficient enough. While the potential benefits of innovative tools and new risk assessment strategies are beyond question, it is essential to further develop the operational applicability of these tools for water management routine. Activities organized by NORMAN have already proven to be effective in laying the ground for the implementation of new strategies into policy.

So far, NORMAN has been strongly involved in issues related to chemicals of emerging concern in the freshwater cycle and the associated EU policies. NORMAN has recently extended its scope with the establishment of a new working group on CECs in the indoor environment. Likewise, there is the potential for new working groups for CECs in the marine or the terrestrial environment. Furthermore, there are new incentives for the integrated assessment and management of chemicals in the environment and human populations. Recently, the European Commission launched the human biomonitoring project HBM4EU. NORMAN has the potential to support integrated approaches for CECs and provide data and knowledge for environmental contaminants to be candidate substances for research in human biomonitoring.

Finally, a global economy results in worldwide exposure to chemical stressors, including CECs. Other countries beyond Europe are interested in the NORMAN activities and Canadian institutions are already partners of the network. Sooner or later, the extension to other regions is likely to become an issue for the NORMAN network.

## Conclusions

After 10 years of activities, NORMAN has become an essential network in support of EU policies. NORMAN integrates EU-wide activities on CECs and facilitates the transfer of the state-of-the-art scientific knowledge to policy makers and regulatory bodies.

Contaminants of emerging concerns are clearly on the EU water policy agenda, e.g., the WFD, and they are also an important issue of chemicals policy, e.g., the legislation for marketing of plant protection products, biocides, and pharmaceuticals. Prioritizing chemicals in the environment for regulation is an increasingly important issue.

Progress in analytical chemistry and increasing monitoring activities reveal the occurrence of a large number of chemical substances in the environment. It is, therefore, necessary to complement the traditional approach for risk assessment with new tools. NORMAN encourages the development of collaborative R&D strategies with a view to their integration into policy. However, new techniques and new monitoring approaches need to prove that they can be used in regulatory routine programs and that they are cost-efficient. As a collaborative and multidisciplinary platform, NORMAN fosters the exchange of information, validation, and harmonization work and helps the achievement of consensus within the wider international community on the implementation of the research results into policy.

The environmental and human exposure to chemicals of emerging concern need to be assessed in a comprehensive way, taking into account all environmental compartments and the impact on human health.

Without the enormous commitment, efforts, and in-kind contributions of the NORMAN members, the NORMAN success stories would not have been possible. It is fully to be expected that this record of success will carry on into the future, as NORMAN’s vision and achievements continue to support EU chemicals management.

## Abbreviations

AFB: Agence Française pour la Biodiversité
ARB: antibiotic-resistant bacteria
ARG: antibiotic-resistant gene
CAS: Chemical Abstracts Service
CEC: contaminants of emerging concern
CEN: European Committee for Standardization
CEN TS: CEN Technical Specifications
CIS: Common Implementation Strategy of the WFD
DG ENV: Directorate General for Environment of the European Commission
DG JRC: Directorate General Joint Research Centre of the European Commission
DG Research: Directorate General for Research and Innovation of the European Commission
DWD: Drinking Water Directive
DWPE: drinking water purification effort
EC: European Commission
ECHA: European Chemicals Agency
EDA: effect-directed analysis
EEA: European Environment Agency
EQS: environmental quality standard
ICPDR: International Commission for the Protection of the Danube River
IPCHEM: European Information Platform for Chemical Monitoring
JDS: Joint Danube Survey
LOQ: limit of quantification
MSFD: Marine Strategy Framework Directive
NTS: non-target screening
PBT: persistent bioaccumulable and toxic
PFAS: polyfluorinated alkyl substances
PMOC: persistent and mobile organic compounds
PNEC: predicted no-effect concentrations
RBSPs: River Basin-Specific Pollutants
SVHC: substances of very high concern
REACH: European Regulation on Registration, Evaluation, Authorisation and Restriction of Chemicals
UBA: Umweltbundesamt (German Federal Environment Agency)
WFD: Water Framework Directive
